# Supplementary material for: Single-cell transcriptome and translatome dual-omics reveals potential mechanisms of human oocyte maturation
Source: Nat Commun. 2022 Aug 30;13:5114. doi: 10.1038/s41467-022-32791-2 (PMC9427852; doi:10.1038/s41467-022-32791-2)
Supplement: Supplementary file 1 — Supplementary Information [file 41467_2022_32791_MOESM1_ESM.pdf]

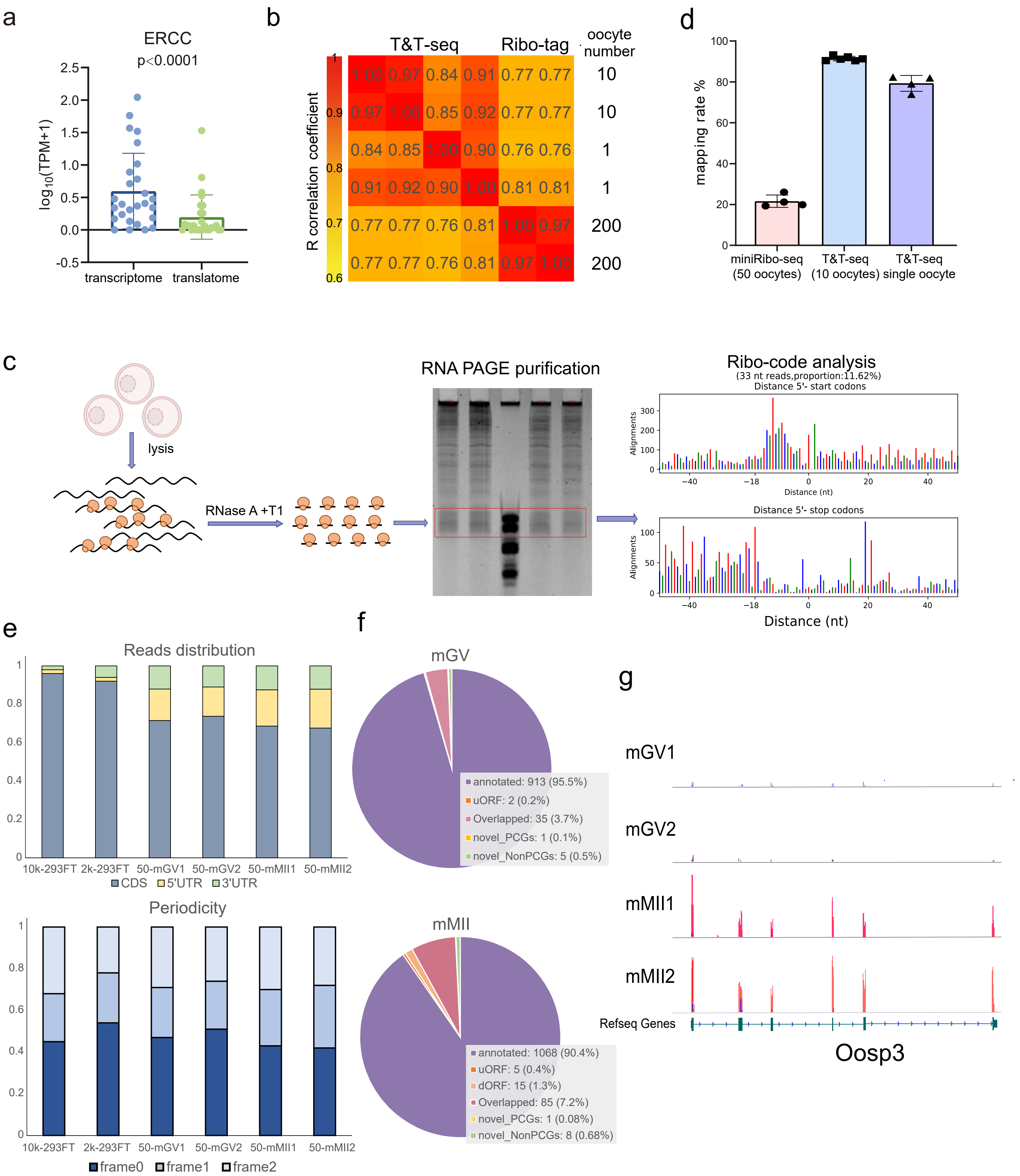
a
ERCC
p<0.0001
log10(TPM+1)
transcriptome
translatome
b
T&T-seq
Ribo-tag
oocyte number
R correlation coefficient
1.00 0.97 0.84 0.91 0.77 0.77 10
0.97 1.00 0.85 0.92 0.77 0.77 10
0.84 0.85 1.00 0.90 0.76 0.76 1
0.91 0.92 0.90 1.00 0.81 0.81 1
0.77 0.77 0.76 0.81 1.00 0.97 200
0.77 0.77 0.76 0.81 0.97 1.00 200
d
mapping rate %
miniRibo-seq (50 oocytes)
T&T-seq (10 oocytes)
T&T-seq single oocyte
c
lysis
RNase A +T1
RNA PAGE purification
Ribo-code analysis
(33 nt reads,proportion:11.62%)
Distance 5'- start codons
Alignments
Distance (nt)
Distance 5'- stop codons
Distance (nt)
e
Reads distribution
10k-293FT 2k-293FT 50-mGV1 50-mGV2 50-mMII1 50-mMII2
CDS 5'UTR 3'UTR
Periodicity
10k-293FT 2k-293FT 50-mGV1 50-mGV2 50-mMII1 50-mMII2
frame0 frame1 frame2
f
mGV
annotated: 913 (95.5%)
uORF: 2 (0.2%)
Overlapped: 35 (3.7%)
novel_PCGs: 1 (0.1%)
novel_NonPCGs: 5 (0.5%)
mMII
annotated: 1068 (90.4%)
uORF: 5 (0.4%)
dORF: 15 (1.3%)
Overlapped: 85 (7.2%)
novel_PCGs: 1 (0.08%)
novel_NonPCGs: 8 (0.68%)
g
mGV1
mGV2
mMII1
mMII2
Refseq Genes
Oosp3

Supplementary Fig.1 Validation experiments for T&T-seq. (a) ERCC spike-in experiment to estimate false positive rate of Ribolace; Each dot represent one ERCC RNA. Data are presented as mean values +/- SD, pair two-tail t-test. (b) Correlation coefficient heatmap of T&T-seq and Ribo-tag (published) sequencing data. (c) Schematic diagram showing the procedures of miniRibo-seq. Single nucleotide resolution of 3-nt periodicity analyzed by RiboCode are shown on the right.Created with BioRender.com. (d) Comparison of mapping rate of miniRibo-seq and T&T-seq. 4 biological replicates in miniRibo-seq, 6 biological replicates in 10 oocytes T&T-seq, 4 biological replicates in single oocyte T&T-seq. error bar, SD. (e) Reads distribution analysis and periodicity analysis of miniRibo-seq of different cell types and cell num-bers. 293FT cells (10,000 and 2,000 cell), mouse oocytes (50 oocytes). (f) ORFs distribution analysis of miniRibo-seq data analyzed by RiboCode. (g) Example of miniRibo-seq read distribution at the mouse Oosp3 gene locus.

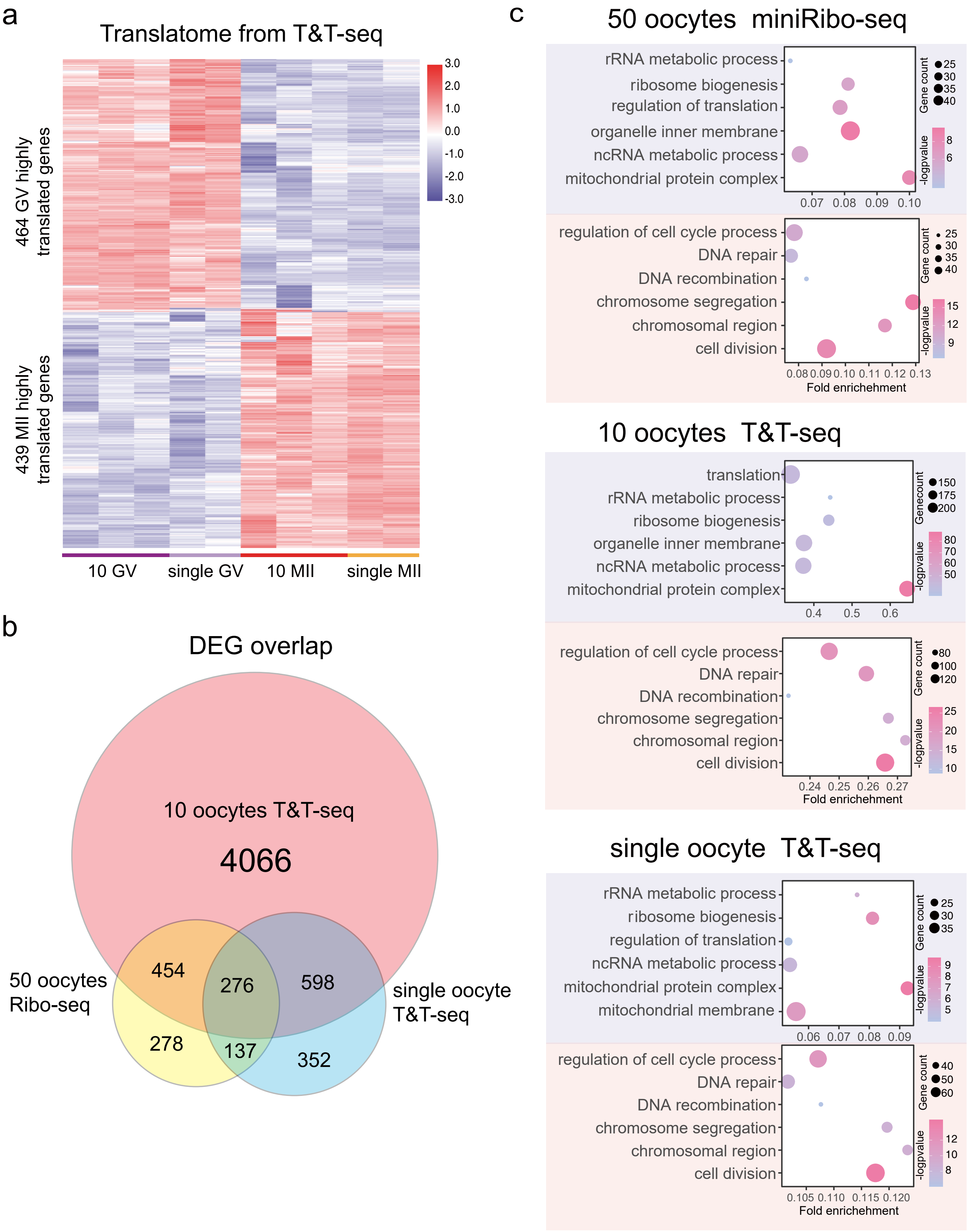
a
Translatome from T&T-seq
464 GV highly translated genes
439 MII highly translated genes
3.0
2.0
1.0
0.0
-1.0
-2.0
-3.0
10 GV
single GV
10 MII
single MII
b
DEG overlap
10 oocytes T&T-seq
4066
50 oocytes Ribo-seq
single oocyte T&T-seq
454
276
598
278
137
352
c
50 oocytes miniRibo-seq
rRNA metabolic process
ribosome biogenesis
regulation of translation
organelle inner membrane
ncRNA metabolic process
mitochondrial protein complex
0.07 0.08 0.09 0.10
Gene count
25
30
35
40
-logpvalue
8
6
regulation of cell cycle process
DNA repair
DNA recombination
chromosome segregation
chromosomal region
cell division
0.08 0.09 0.10 0.11 0.12 0.13
Fold enrichehment
15
12
9
10 oocytes T&T-seq
translation
rRNA metabolic process
ribosome biogenesis
organelle inner membrane
ncRNA metabolic process
mitochondrial protein complex
0.4 0.5 0.6
Genecount
150
175
200
80
70
60
50
regulation of cell cycle process
DNA repair
DNA recombination
chromosome segregation
chromosomal region
cell division
0.24 0.25 0.26 0.27
Fold enrichehment
80
100
120
25
20
15
10
single oocyte T&T-seq
rRNA metabolic process
ribosome biogenesis
regulation of translation
ncRNA metabolic process
mitochondrial protein complex
mitochondrial membrane
0.06 0.07 0.08 0.09
25
30
35
9
8
7
6
5
regulation of cell cycle process
DNA repair
DNA recombination
chromosome segregation
chromosomal region
cell division
0.105 0.110 0.115 0.120
Fold enrichehment
40
50
60
12
10
8

Supplementary Fig.2 Comparisons of mouse-oocyte translatomes produced by different methods. (a) Expression heatmap of DEGs during mouse oocyte maturation identified by T&T-seq (10 and single oocyte) $p < 0.05$, Wald test, log2 foldchange >1. (b) Venn plot showing the overlap of DEGs between 10 oocytes T&T-seq, single oocytes T&T-seq and 50 oocytes miniRibo-seq. (c) Representative GO enrichment of upregulated DEGs identi-fied by miniRibo-seq (50 oocytes) and T&T-seq(10 or single oocyte). Blue background covers terms enriched in GV oocytes, red background covers MII oocytes (p value, hypergeometric test).

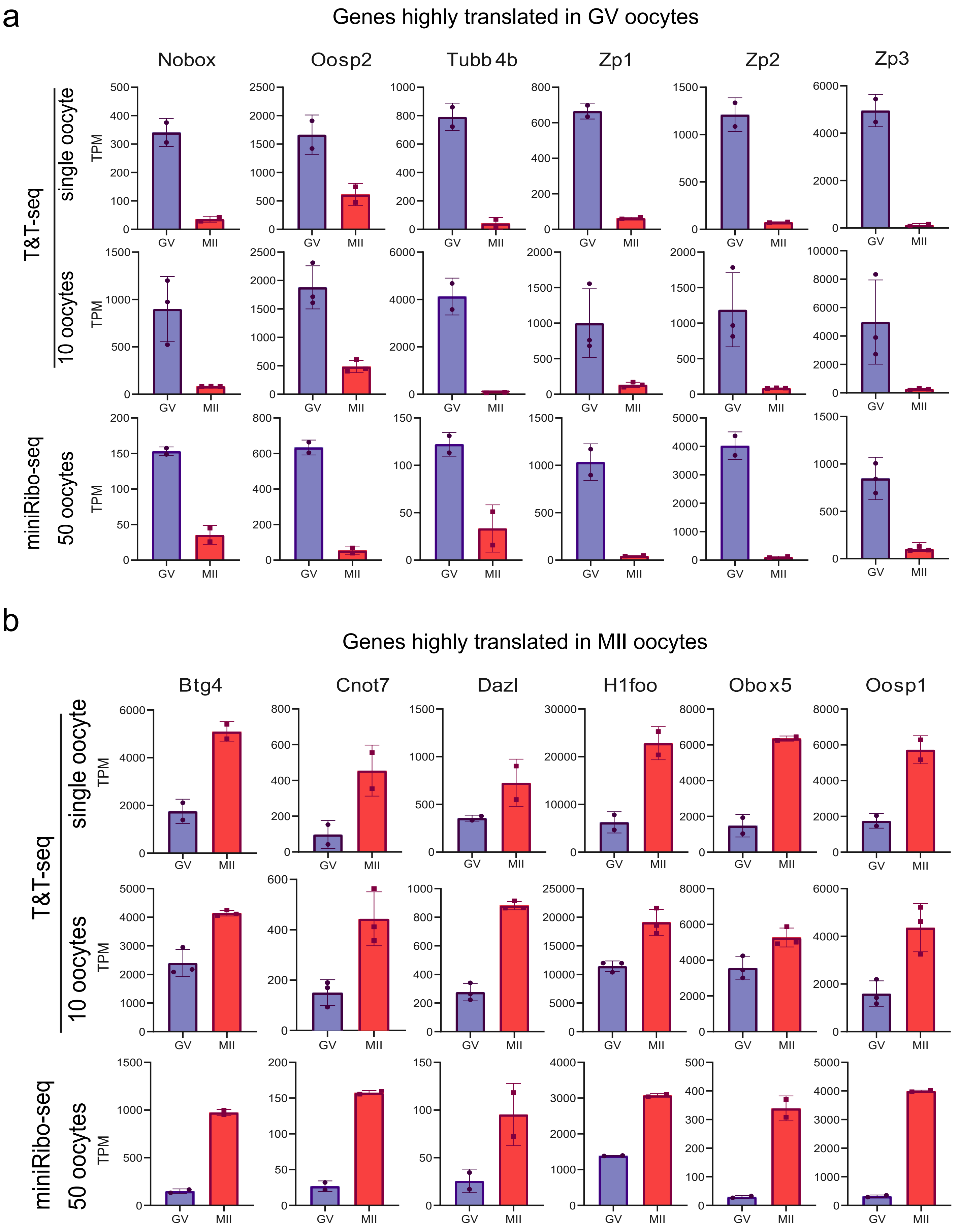
a
Genes highly translated in GV oocytes
Nobox
Oosp2
Tubb4b
Zp1
Zp2
Zp3
T&T-seq
single oocyte
10 oocytes
miniRibo-seq
50 oocytes
TPM
GV
MII
b
Genes highly translated in MII oocytes
Btg4
Cnot7
Dazl
H1foo
Obox5
Oosp1
T&T-seq
single oocyte
10 oocytes
miniRibo-seq
50 oocytes
TPM
GV
MII

Supplementary Fig.3 Translational expression levels of T&T-seq and miniRibo-seq of genes highly trans- lated in GV and MII oocytes. (a) Comparison of translational expres-sion levels of T&T-seq and miniRibo-seq of genes highly translated in GV oocytes. (b) Comparison of translational expression levels of T&T-seq and miniRibo-seq of genes highly translated in MII oocytes. 2 biological replicates in miniRibo-seq, 3 biological repli-cates in 10 oocytes T&T-seq, 2 biological replicates in single oocyte T&T-seq. Data are presented as mean values +/- SD.

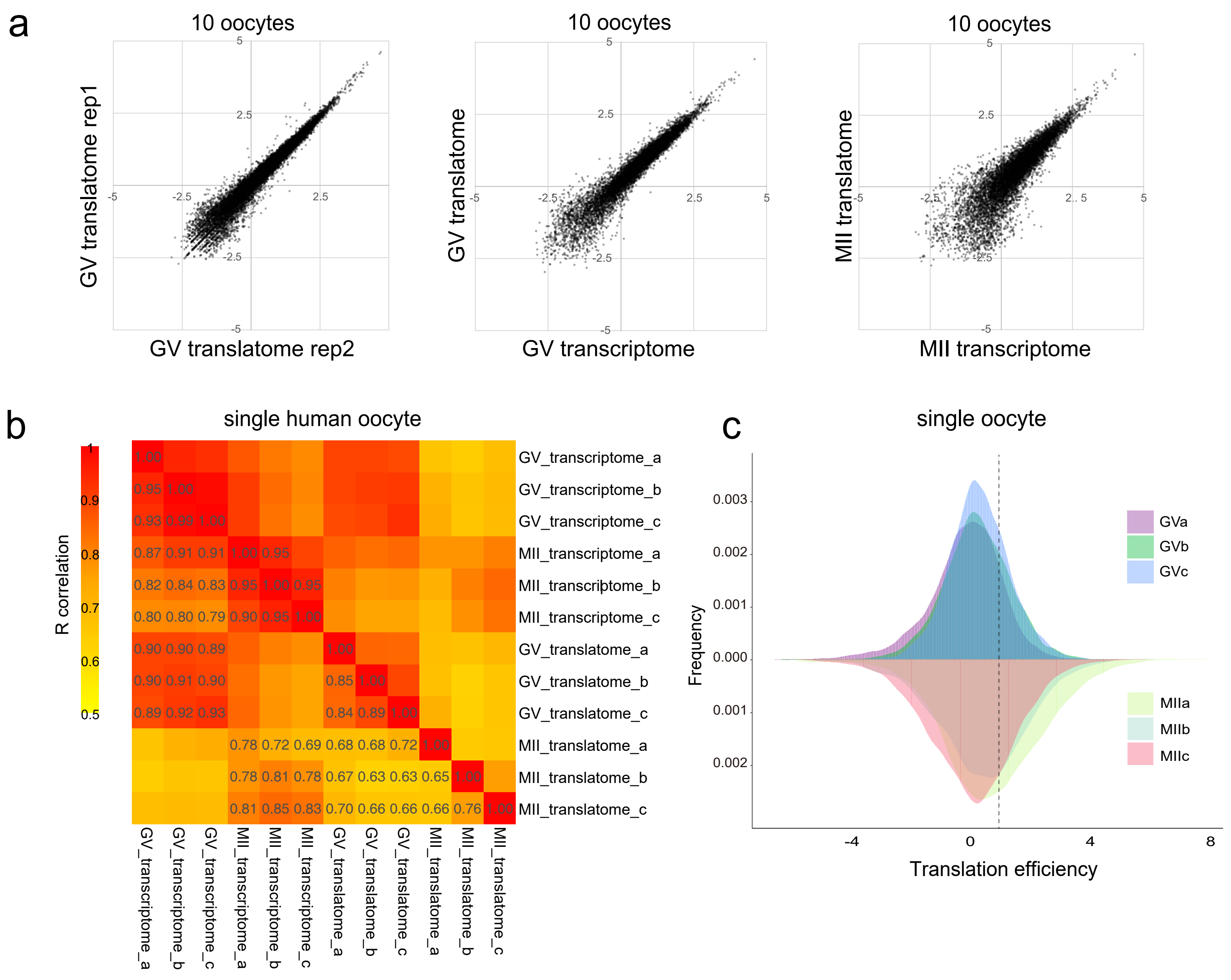

Supplementary Fig.4 T&T variability and diversity in human GV and MII oocytes. (a) Scatter plots showing the expression of transcriptome versus translatome in 10 oocyte samples. Genes expression are represented by $\log_2$(TPM+1). (b) Correlation coefficient heatmap showing the correlations of transcription and translation among single human GV or MII oocytes. (c) Frequency distribution histogram of the translation efficiency of single human oocytes. Upper: GV oocytes. Lower: MII oocytes.

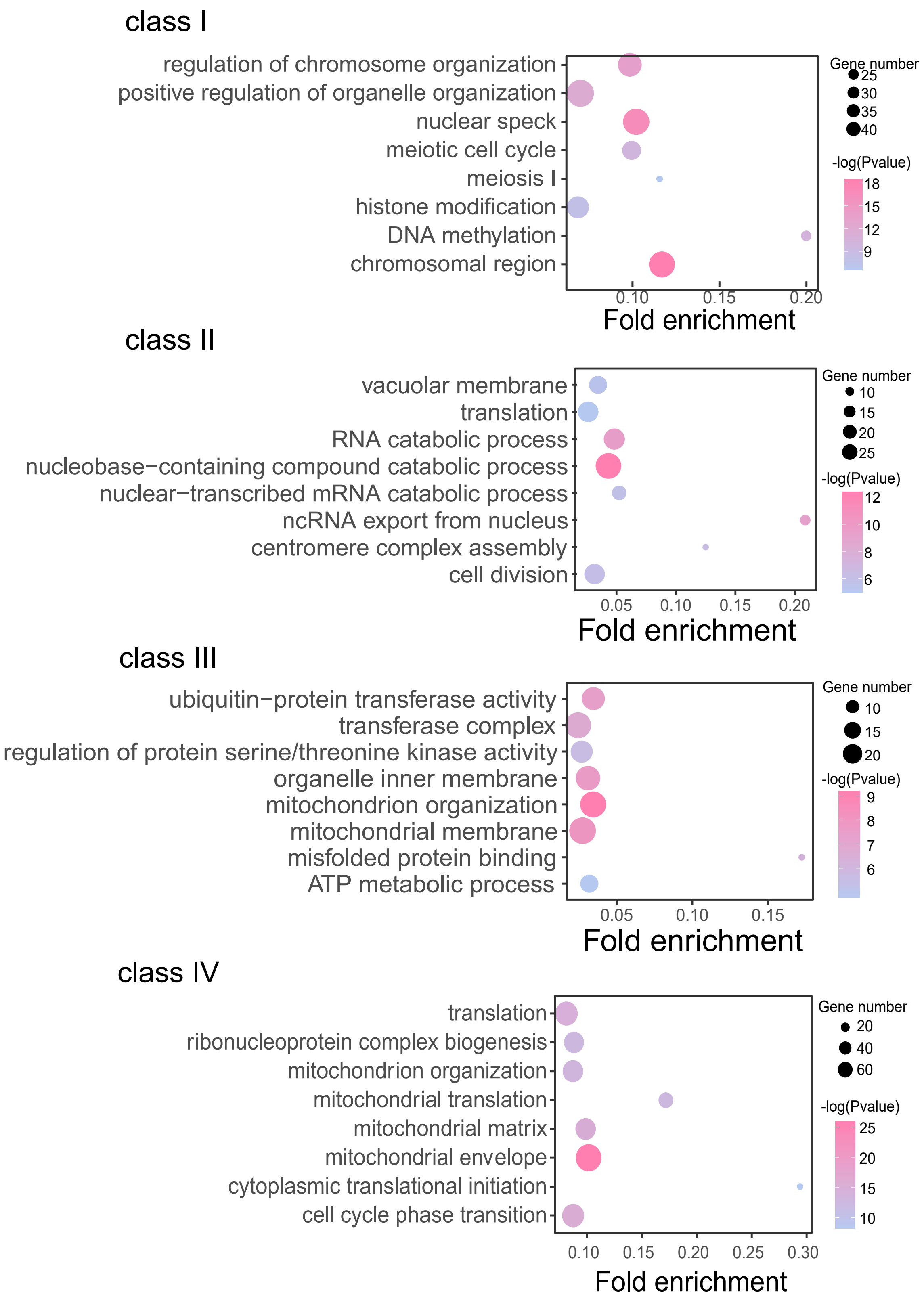

Supplementary Fig.5 Representative GO terms of different classes of genes according to the T&T analysis of human GV versus MII oocytes. (p value, hypergeometric test)

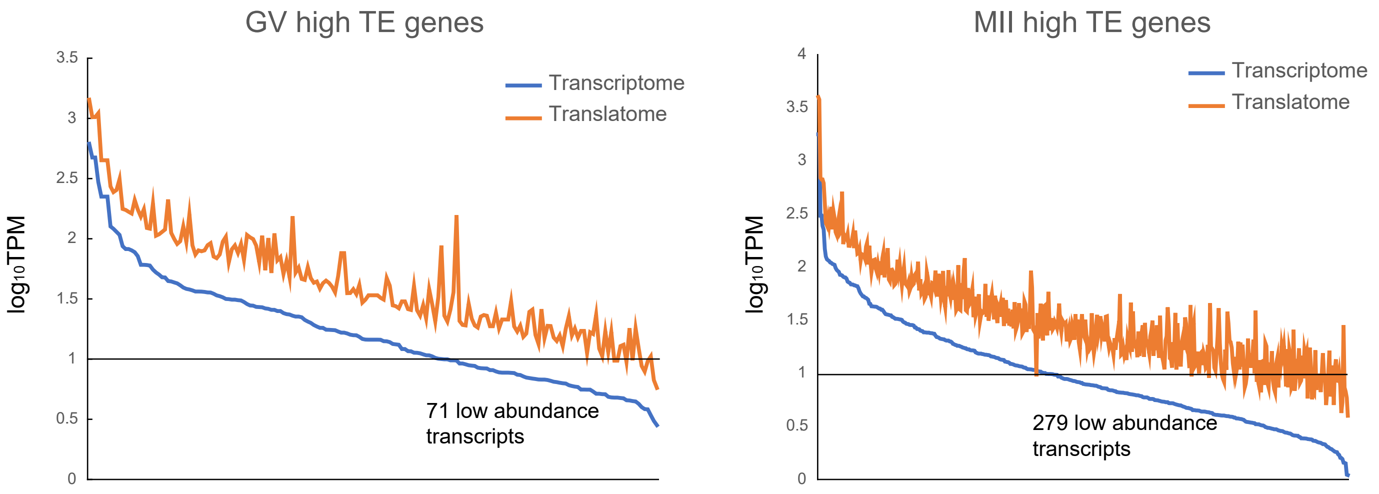

Supplementary Fig.6 Abundance analysis of high TE genes in human GV and MII oocytes.

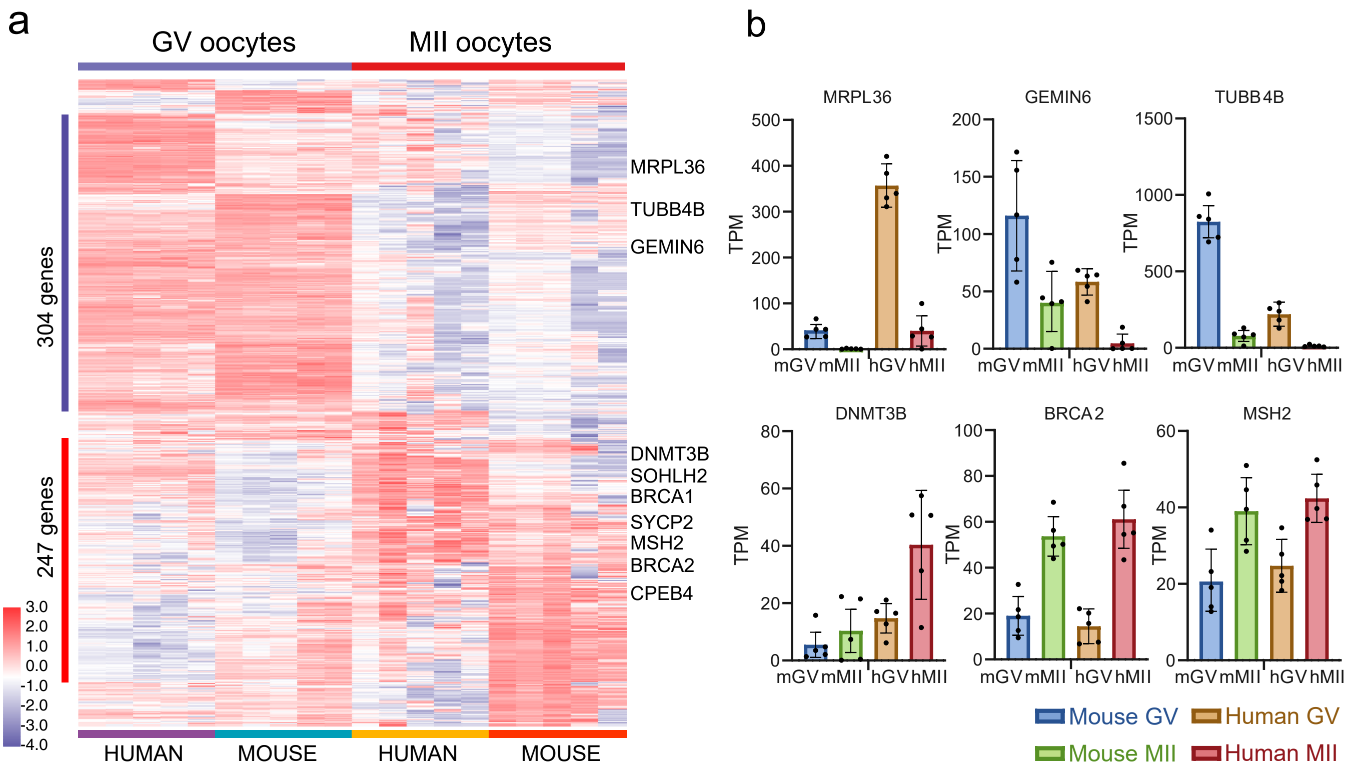

Supplementary Fig.7 Translatome consistency between human and mouse oocytes. (a) Representative gene expression heatmap of unique translated DEGs based on human expression patterns. Selective human MII enriched genes are labeled on the right. (b) Translational expression levels of the representative genes of human and mouse GV and MII oocytes. 5 biological replicates of human and mouse oocytes. Data are presented as mean values +/- SD, Unpaired two-sided t-test.

RBP name	Motif	Log_2 FC	p value
SRSF2	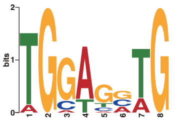	1.8365	3.98E-12
nElavl	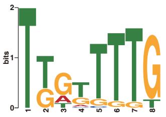	1.65577	6.37E-09
SRSF4	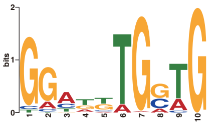	1.60991	4.17E-07
CELF4	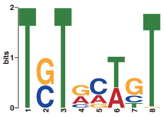	1.48712	7.37E-07
RBFOX2	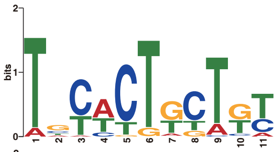	1.29572	1.08E-06
CELF	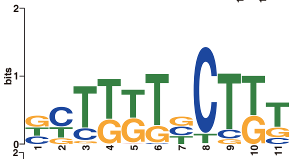	1.21587	1.67E-08
SRSF1	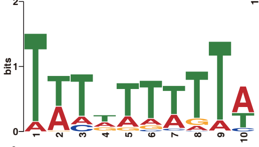	1.17189	1.89E-12
FMR1	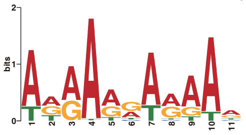	1.15369	2.28E-07
CPSF6	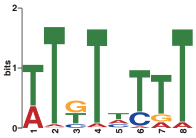	1.05479	2.71E-08
EZH2	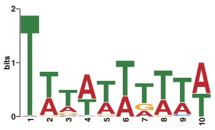	1.01599	2.02E-09

Supplementary Fig.8 Putative RBPs and their 3’UTR binding motifs enriched in the of high TE genes in mouse MII oocytes. One-sided t-test.

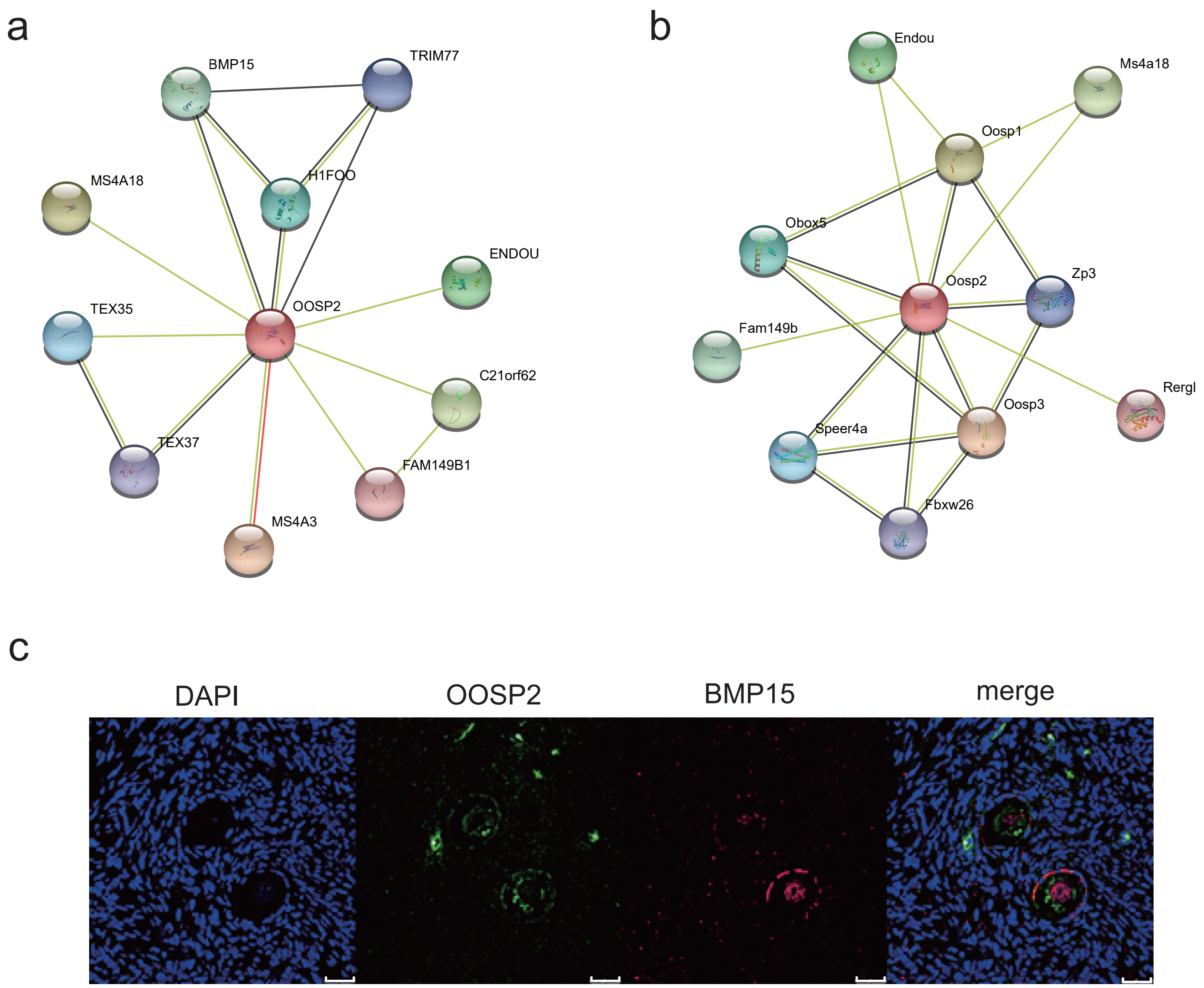

Supplementary Fig.9 Protein-protein interaction analysis (STRING analysis) and immunostaining of OOSP2 in human ovary. (a) Network of human OOSP2. (b) Network of mouse Oosp2. c Immunofluorescence staining of human ovary with OOSP2 (1:200) and BMP15 (1:200) antibody. Two independent experiments show the same result.

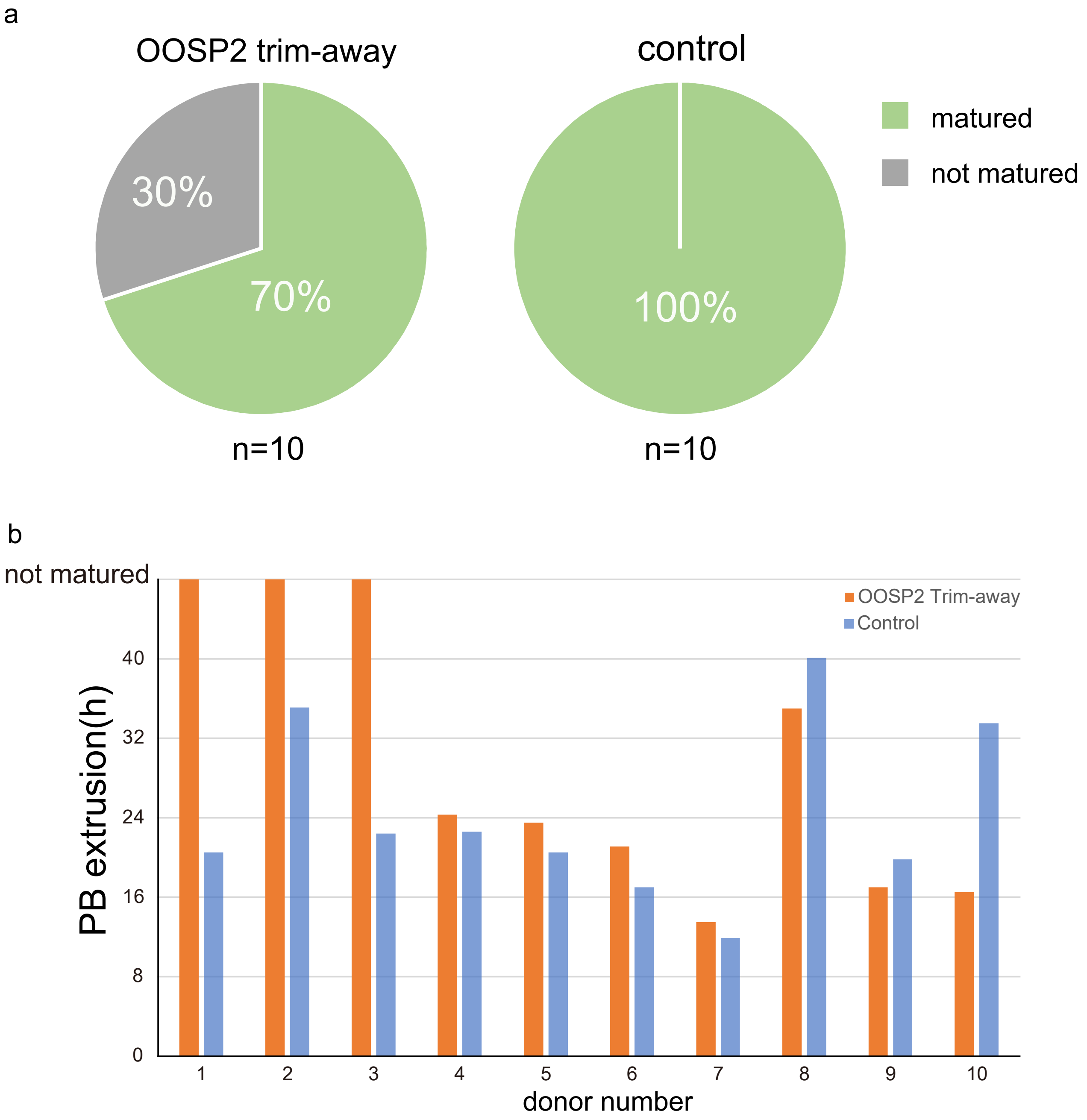

Supplementary Fig.10 Validating OOSP2 maturation effect on human GV oocytes using TRIM-away methods. (a) Maturation rate of human GV oocytes by timelapse observation between OOSP2 Trim-away group and control group. Comparison was conducted by using pairwise GV oocytes from the same donors. (b) Comparison of PBE (PB exclusion) of OOSP2 Trim-away group and control group.

a

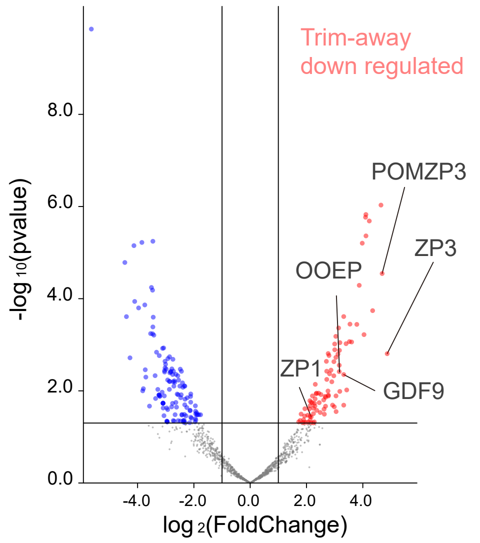

b

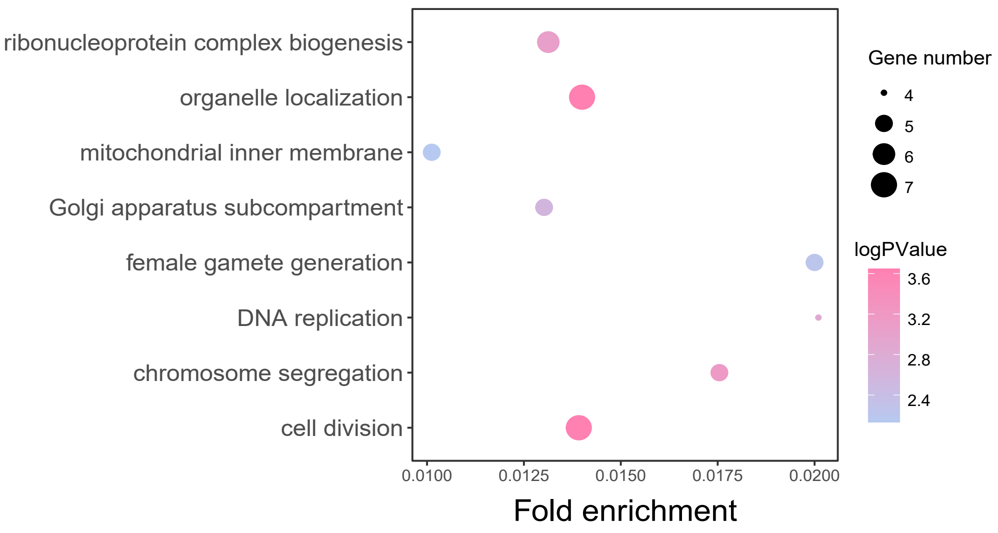

Supplementary Fig.11 Translatome DEGs of OOSP2 Trim-away versus control oocytes. (a) Volcano plot showing the DEGs after OOSP2 Trim-away. Some downregulated genes are highlighted, including ZP1, GDF9, OOEP, ZP3, and POMZP3 (p value, Wald test). (b) GO analysis showing the terms the enriched down-regulated genes after OOSP2 Trim-away. (p value, hypergeometric test).

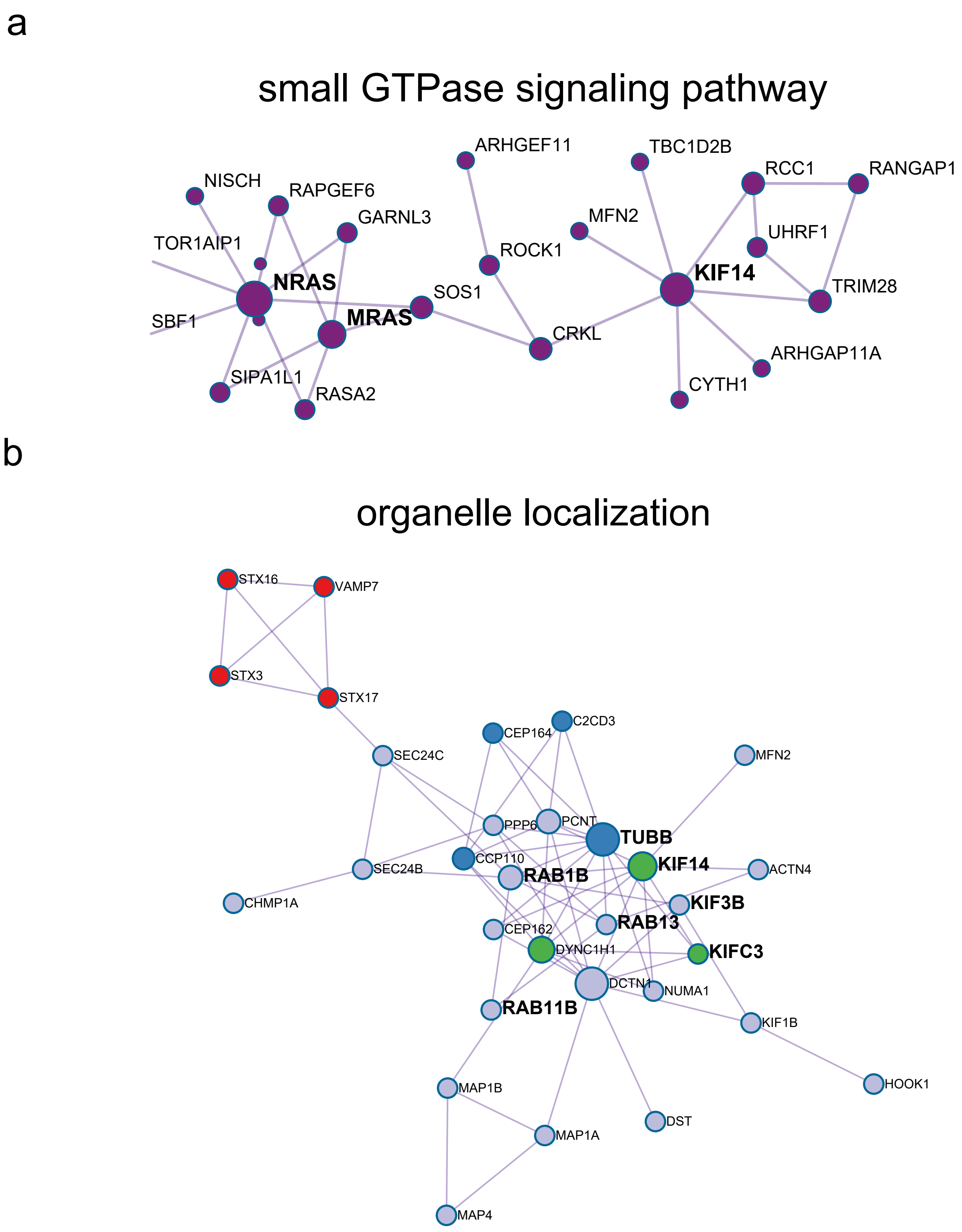

Supplementary Fig.12 Protein-protein interaction network analysis of genes upregulated by recombinant OOSP2 in human GV oocytes. (a) “small GTPase signaling pathway”, (b) “organelle localization”. Colors represents different MCODEs (see methods).
