# Supplementary material for: Single-cell transcriptome and translatome dual-omics reveals potential mechanisms of human oocyte maturation
Source: Nat Commun. 2022 Aug 30;13:5114. doi: 10.1038/s41467-022-32791-2 (PMC9427852; doi:10.1038/s41467-022-32791-2)
Supplement: Supplementary file 3 — Description of Additional Supplementary Files [file 41467_2022_32791_MOESM3_ESM.pdf]

Description of Additional Supplementary Files

Supplementary Data 1
Description: T&T-seq data of immature and matured mouse oocytes. 3 biological replicates for 10 oocytes and 2 biological replicates for single oocyte.

Supplementary Data 2
Description: Sequence information of miniRibo-seq and T&T-seq for human and mouse oocytes.

Supplementary Data 3
Description: GO enrichment results of DEGs identified by miniRibo-seq and T&T-seq during mouse oocyte maturation.

Supplementary Data 4
Description: T&T-seq data of immature and matured human oocytes. 2 biological replicates for 10 oocytes and 3 biological replicates for single oocyte.

Supplementary Data 5
Description: High translation efficiency genes during human oocyte maturation identified by 10 oocytes T&T-seq data.

Supplementary Data 6
Description: GO enrichment results of DEGs of high translation efficiency genes during human oocyte maturation identified by 10 oocytes T&T-seq data

Supplementary Data 7
Description: RBP motifs enriched in the 3’UTR of high translation efficiency genes during human oocyte maturation identified by 10 oocytes T&T-seq data

Supplementary Data 8
Description: Human unique genes compared with mouse using T&T-seq data.

Supplementary Data 9
Description: GO enrichment results of human unique genes compared with mouse using T&T-seq data.

Supplementary Data 10
Description: RBP motifs enriched in the 3’UTR of high translation efficiency genes during mouse oocyte maturation identified by 10 oocytes T&T-seq data

Supplementary Data 11
Description: Expression of secreted proteins identified in human oocyte maturation.

Supplementary Data 12

Description: T&T-seq data of human oocytes IVM experiments. 4 biological replicates for GV control oocytes and 4 biological replicates for hRec-OOSP2 treated oocytes and 6 biological replicates for OOSP2 antibody treated oocytes.

Supplementary Data 13

Description: Primer sequence for dual-luciferase experiment.
