# Supplementary material for: Single-cell transcriptome and translatome dual-omics reveals potential mechanisms of human oocyte maturation
Source: Nat Commun. 2022 Aug 30;13:5114. doi: 10.1038/s41467-022-32791-2 (PMC9427852; doi:10.1038/s41467-022-32791-2)
Supplement: Supplementary file 17 — Reporting Summary [file 41467_2022_32791_MOESM17_ESM.pdf]

nature portfolio

Corresponding author(s): Kee, Kehkooi

Last updated by author(s): Jul 25, 2022

Reporting Summary

Nature Portfolio wishes to improve the reproducibility of the work that we publish. This form provides structure for consistency and transparency in reporting. For further information on Nature Portfolio policies, see our Editorial Policies and the Editorial Policy Checklist.

Statistics

For all statistical analyses, confirm that the following items are present in the figure legend, table legend, main text, or Methods section.

n/a	Confirmed	
☐	☒	The exact sample size (*n*) for each experimental group/condition, given as a discrete number and unit of measurement
☐	☒	A statement on whether measurements were taken from distinct samples or whether the same sample was measured repeatedly
☐	☒	The statistical test(s) used AND whether they are one- or two-sided<br>*Only common tests should be described solely by name; describe more complex techniques in the Methods section.*
☐	☒	A description of all covariates tested
☐	☒	A description of any assumptions or corrections, such as tests of normality and adjustment for multiple comparisons
☐	☒	A full description of the statistical parameters including central tendency (e.g. means) or other basic estimates (e.g. regression coefficient) AND variation (e.g. standard deviation) or associated estimates of uncertainty (e.g. confidence intervals)
☐	☒	For null hypothesis testing, the test statistic (e.g. F, t, r) with confidence intervals, effect sizes, degrees of freedom and P value noted<br>*Give P values as exact values whenever suitable.*
☒	☐	For Bayesian analysis, information on the choice of priors and Markov chain Monte Carlo settings
☒	☐	For hierarchical and complex designs, identification of the appropriate level for tests and full reporting of outcomes
☐	☒	Estimates of effect sizes (e.g. Cohen's d, Pearson's r), indicating how they were calculated

Our web collection on statistics for biologists contains articles on many of the points above.

Software and code

Policy information about availability of computer code

Data collection	For high-throughput sequencing data collection, we used Nova 6000 platform.
Data analysis	For statistical analyses, we used R (R studio version 1.1.456), GraphPad Prism 6 and TBTools (v1.09854).<br>For high throughput sequencing data processing and subsequent data analyses we used:<br>Trim_galore (v0.6.4)<br>Hisat2 (v2.1.0)<br>Featurecounts (v1.6.5)<br>DESeq2 (v1.26.0)<br>Metascape<br>Seurat (3.2.3)<br>Monocle (v2.10.1)<br>Cutadapt (v1.18)<br>Bowtie2(v2.3.5.1)<br>STAR (v2.7.1a)<br>Ribocode (1.2.11)<br>MEME-suite(5.4.1)<br>Motif enrichment scripts: https://github.com/lynhsiong/MotifEnrichForUTR.

For manuscripts utilizing custom algorithms or software that are central to the research but not yet described in published literature, software must be made available to editors and reviewers. We strongly encourage code deposition in a community repository (e.g. GitHub). See the Nature Portfolio guidelines for submitting code & software for further information.

Data

Policy information about availability of data

All manuscripts must include a data availability statement. This statement should provide the following information, where applicable:

- Accession codes, unique identifiers, or web links for publicly available datasets
- A description of any restrictions on data availability
- For clinical datasets or third party data, please ensure that the statement adheres to our policy

The sequencing data generated in this study have been deposited in the Gene Expression Omnibus (GEO) under accession number GSE197578, and are publicly available. mm10 genome and hg38 genome were download from Gencode (https://www.gencodegenes.org/).

Field-specific reporting

Please select the one below that is the best fit for your research. If you are not sure, read the appropriate sections before making your selection.

☒ Life sciences ☐ Behavioural & social sciences ☐ Ecological, evolutionary & environmental sciences

For a reference copy of the document with all sections, see nature.com/documents/nr-reporting-summary-flat.pdf

Life sciences study design

All studies must disclose on these points even when the disclosure is negative.

Sample size	For T&T-seq, we use 10 oocytes or single oocyte in one sample. For miniRibo-seq, we use 50 mouse oocytes in one sample. The choice of sample size refers to similar publications, Ribotag (Luong et al, 2020) and liRibo-seq (Zhang et al, 2022).
Data exclusions	No data were excluded from the analysis.
Replication	2 biological replicates for 10 human oocytes sample. 3 biological replicates for single human oocyte sample. 3 biological replicates for 10 mouse oocytes sample. 2 biological replicates for single mouse oocyte sample. 4 biological replicates for single human oocyte treated by OOSP2 protein. 24 biological replicates for hRecOOSP2 IVM experiment. 16 biological replicates for OOSP2 anti body IVM experiment. 10 biological replicates for OOSP2 Trim-away IVM experiment. The significance of the experimental replications were confirmed by statistical analysis indicated in the figure legends.
Randomization	Oocytes used in experiments were collected randomly from donors younger than 35 years old. Samples were allocated into experimental groups randomly.
Blinding	Researchers were blind to group allocation.

Reporting for specific materials, systems and methods

We require information from authors about some types of materials, experimental systems and methods used in many studies. Here, indicate whether each material, system or method listed is relevant to your study. If you are not sure if a list item applies to your research, read the appropriate section before selecting a response.

Materials & experimental systems

n/a	Involved in the study	
☐	☒	Antibodies
☐	☒	Eukaryotic cell lines
☒	☐	Palaeontology and archaeology
☐	☒	Animals and other organisms
☐	☒	Human research participants
☒	☐	Clinical data
☒	☐	Dual use research of concern

Methods

n/a	Involved in the study	
☒	☐	ChIP-seq
☒	☐	Flow cytometry
☒	☐	MRI-based neuroimaging

Antibodies

Antibodies used	OOSP2(TMEM122), Abcam,ab187749. This antibody has been claimed to react with human by the manufacturer and was used for IHC-P， used as 1:200 dilution.<br>Normal Rabbit IgG, Millipore,12-370; RRID:AB_145841.<br>BMP15(GDF-9B),SANTA CRUZ,sc-271824;RRID:AB_10708552, used as 1:200 dilution.
Validation	OOSP2(TMEM122), Abcam,ab187749. This antibody has been claimed to react with human by the manufacturer and was used for

Validation	IHC-P.<br>Anti-GDF-9B Antibody (F-7) is recommended for detection of mature and precursor GDF-9B of human and hamster origin by WB, IP, IF, IHC(P) and ELISA

Eukaryotic cell lines

Policy information about cell lines

Cell line source(s)	Human: 293FT cells were purchased from Thermo Fisher Scientific (Cat#R70007).
Authentication	293FT cells is a commercial cell line from Thermo Fisher Scientific.
Mycoplasma contamination	All cells have been tested to be negative of Mycoplasma.
Commonly misidentified lines (See ICLAC register)	All cells are not in the ICLAC database.

Animals and other organisms

Policy information about studies involving animals; ARRIVE guidelines recommended for reporting animal research

Laboratory animals	We used 4 weeks B6/C57 female mouse for mouse oocytes collection. The mouse facility maintains 12 hour/12 hour of dark/light cycle, 20-26 degree Celsius and 40-70% humidity.
Wild animals	This study does not contain any wild animals.
Field-collected samples	This study does not contain any field-collected samples.
Ethics oversight	All animal maintenance and experimental procedures were performed according to the guidelines of the Institutional Animal Care and Use Committee (IACUC) of Tsinghua University, Beijing, China. Director: Hongwei Wang; Chair： Hai Qi; Veterinarian: Zhongchen Xie; Board members: Yiguo Wang, Gong Chen, Xiaoyu Wu.

Note that full information on the approval of the study protocol must also be provided in the manuscript.

Human research participants

Policy information about studies involving human research participants

Population characteristics	Donated oocytes and embryos were collected with patients' consent from younger than 35 years old from the Sixth Affiliated hospital of Sun Yat-Sen University, the Provincial Hospital Affiliated to Shandong University, the First Affiliated Hospital of Nanjing Medical University, the First Affiliated Hospital of Wenzhou Medical University, and the Shanghai Tenth People's Hospital of Tongji University. Patients underwent ART treatment from July 2017 to March 2021 were enrolled.
Recruitment	Donors are recruited voluntarily. Donated oocytes and embryos were collected with patients' consent and was approved by the ethical committee of the Sixth Affiliated hospital of Sun Yat-Sen University, the Provincial Hospital Affiliated to Shandong University, the First Affiliated Hospital of Nanjing Medical University, the First Affiliated Hospital of Wenzhou Medical University, and the Shanghai Tenth People's Hospital of Tongji University. Patients underwent ART treatment from July 2017 to March 2021 were enrolled. Conventional protocols, including the antagonist and the long agonist protocol were applied for controlled ovarian hyperstimulation (COH). Transvaginal ultrasound-guided oocyte retrieval was performed 36 h (±2) after hCG trigger.
Ethics oversight	Project was approved by the ethical committee of the Sixth Affiliated hospital of Sun Yat-Sen University

Note that full information on the approval of the study protocol must also be provided in the manuscript.
